# Disturbed relationship between glucocorticoid receptor and 5-HT1AR/5-HT2AR in ADHD rats: A correlation study

**DOI:** 10.3389/fnins.2022.1064369

**Published:** 2023-01-09

**Authors:** Xiaoxia Lin, Liang Huang, Huifang Huang, Zhongling Ke, Yanhui Chen

**Affiliations:** Department of Pediatrics, Fujian Medical University Union Hospital, Fuzhou, China

**Keywords:** attention deficit hyperactivity disorder, glucocorticoid receptor, 5-HT, 5-HT1A receptor, 5-HT2A receptor

## Abstract

**Objective:**

This work is to investigate the alterations of the central 5-hydroxytryptamine (5-HT) system in spontaneously hypertensive rats (SHR) and the correlation with the behaviors of SHR, and to explore the effects of glucocorticoid intervention on the central 5-HT system and SHR behaviors.

**Materials and methods:**

Three weeks old SHR were chosen as the attention-deficit hyperactivity disorder (ADHD) model and treated with glucocorticoid receptor (GR) agonist or inhibitor, whereas Wista Kyoto rats (WKY) were chosen as the normal control group. Open-field test and Làt maze test were used to evaluate the spontaneous activities and non-selective attention. The levels of 5-HT in the extracellular fluid specimens of the prefrontal cortex of rats were analyzed by high-performance liquid chromatography. The expressions of GR, 5-HT1A receptor (5-HT1AR), and 5-HT2A receptor (5-HT2AR) in the prefrontal cortex were analyzed through immunohistochemistry.

**Results:**

Our study demonstrated that the 5-HT level was lower in the prefrontal cortex of SHR compared to that of WKY. The Open-field test and Làt maze test showed that GR agonist (dexamethasone, DEX) intervention ameliorated attention deficit and hyperactive behavior, whereas GR inhibitor (RU486) aggravated the disorders. With DEX, the expression levels of 5-HT and 5-HT2AR in the prefrontal cortex of SHR were significantly higher than those in the control group, whereas the expression level of 5-HT1AR was lower. However, the expression levels of 5-HT and 5-HT2AR were significantly decreased after the intervention with RU486, while the expression level of 5-HT1AR increased. Results showed that glucocorticoid was negatively correlated with 5-HT1AR and positively correlated with 5-HT2AR.

**Conclusion:**

In the prefrontal cortex of ADHD rats, the down-regulation of 5-HT and 5-HT2AR expressions and the up-regulation of 5-HT1AR, compared with WYK rats, suggested a dysfunctional central 5-HT system in ADHD rats. The GR agonist can upregulate the expression of 5-HT and 5-HT2AR and downregulate the expression of 5-HT1AR in the prefrontal cortex of SHR as well as reduce the hyperactivity and attention deficit behavior in SHR, while the opposite was true for the GR inhibitor. It is suggested that the dysfunction of the 5-HT system in ADHD rats is closely related to glucocorticoid receptor activity.

## 1. Introduction

Attention deficit hyperactivity disorder (ADHD) is the most common neuropsychological and developmental-behavioral disorder in childhood, characterized by a persistent pattern of inattention, hyperactivity, and impulsivity (Posner et al., 2020). The prevalence of school-age children is as high as 5.8% (Sayal et al., 2018). 60 to 85% of those diagnosed in childhood keep having the disorders in their adolescence, and up to 60% of those persist into adulthood (Fayyad et al., 2017). ADHD with those various symptoms negatively impacts individuals’ personal, social, academic, and occupational functioning and development, and increases the burden on the healthcare system (Luo et al., 2019). The high prevalence of ADHD and the great danger it poses to patients and society have made the investigation of its etiology and pathogenesis a hot spot for scholars worldwide.

Interaction of genetic and environmental factors is therefore thought to play a role in the development and precipitation of ADHD. Stress, as one of the aversive environmental factors, has been found to activate the hypothalamic-pituitary-adrenal (HPA) axis to increase the circulating level of glucocorticoids (GCs) (e.g., cortisol in humans) (Corominas et al., 2012; Haleem and Gul, 2020). However, we and some other research groups have found a low level of plasma cortisol in ADHD children, indicating the hypo-responsiveness of the HPA axis in ADHD children and suggesting the close relationship between cortisol and the symptoms of attention deficit, hyperactivity, and impulsive behavior (Ma et al., 2011; Pinto et al., 2016; Angeli et al., 2018). This finding suggested a low level of cortisol may increase the risk of the onset of ADHD. Cortisol exerts its effects through two related receptors: glucocorticoid receptor (GR) and mineralocorticoid receptor (MR) (de Kloet et al., 2019). GRs are widely expressed in the hypothalamus, pituitary gland, and hippocampus, which play a role in regulating cortisol levels during stress state and peak circadian rhythm, while MRs are restricted to limbic areas and are important for maintaining non-stress state and basal cortisol level (Derijk et al., 2008). Joëls (2018) found that the abnormal expressions of GC and GR in the central nervous system were associated with behavioral disorders including emotional change and cognitive activity. The GR plays a pivotal role in the brain’s response to stress; a haplotype of functional polymorphisms in the Nr3C1 gene encoding this receptor has been associated with ADHD (Erickson et al., 2003). Differential activities of the GR and its main endogenous agonist cortisol have been associated with attention, arousal, perception, memory, and emotional processing functions that are frequently impaired in individuals with ADHD (Corbett and Glidden, 2000; Shaw et al., 2014; Talbot and Kerns, 2014). Our early research also found that the expression level of hippocampal GR in the ADHD animal model was significantly lower than that in the control group, while the MR expression level was unchanged. It was suggested that the low expression of GR may be one of the factors leading to the hypo-responsiveness of the HPA axis in the ADHD model under stress (Chen et al., 2017).

The monoamine neurotransmitter metabolic disorder theory is the most important neurobiochemical hypothesis for the pathogenesis of ADHD (Curatolo et al., 2009). A collection of evidence has revealed that ADHD is associated with monoamine neurotransmitters, like serotonin (5-HT), dopamine (DA), and noradrenaline (NE) (Mogavero et al., 2018). 5-HT system plays a vital role in ADHD from genetics, biochemistry, nerve pathology, and psychological perspectives (Riley and Overton, 2019). Furthermore, the prefrontal cortex (PFC) serves as the chief executive officer of the brain, controlling the highest level of cognitive and emotional processes, which are commonly disrupted in mental illnesses, such as ADHD (Hiser and Koenigs, 2018; Yan and Rein, 2022). Many studies of ADHD have reported volumetric abnormalities in PFC and indicated that ventromedial prefrontal cortex structure is a biomarker for ADHD symptomatology (Albaugh et al., 2017; Bayard et al., 2020; Restrepo-Lozano et al., 2022). Aberrant serotonergic neurotransmission has long been implicated in the pathogenesis of neuropsychiatric disorders associated with PFC dysfunction, including schizophrenia, depression, and anxiety (Leonardo and Hen, 2006). Stocker et al. (2003) found that the 5-HT level was reduced in the brains of ADHD animals. And, the high dose of fluvoxamine, a selective 5-HT reuptake inhibitor, significantly inhibited hyperactive behavior, while the low dose had an ameliorating effect on impulsive symptoms. Although it is clear that serotonin plays an important modulatory role in mPFC function, it is far from clear how and through which receptors serotonin exerts these effects. The pleiotropic functions of serotonin are afforded by the concerted actions of multiple serotonin receptor subtypes (Yan and Rein, 2022). 5-HT1A receptor (5-HT1AR), and 5-HT2A receptor (5-HT2AR) are two essential 5-HT receptors, which have been confirmed to be closely related to ADHD associated behaviors (Gongliang and Robert, 2015; Albert et al., 2019). van der Meer et al. (2014) reported that individuals carrying the short variant (S-allele) of a polymorphism in the promoter region of the serotonin transporter gene showed a stronger relationship between stress exposure and ADHD severity than those homozygous for the long variant (L-allele). Administration of dexamethasone, a GR-specific glucocorticoid, increased the expression of the serotonin transporter (5-HTT) (Glatz et al., 2003), and the high availability of genetically conveyed 5-HTT was associated with the lower Nr3C1 expression after stress exposure in rats (van der Meer et al., 2014). Since the interplay between these two receptor types is a key factor in serotonin modulation of cortical function, in this work, we decided to evaluate whether or not the 5-HT1AR and 5-HT2AR are also involved in the regulation of GR in ADHD.

5-HT1A receptor (5-HT1AR) is associated with anxiety, alcohol dependence, and impulsive behavior, and the polymorphism of 5-HT1AR gene can increase the susceptibility of ADHD (Pehrson et al., 2013). 5-HT2AR can regulate the drug-induced release of dopamine (dopamine, DA) and has an impact on hyperactive, impulsive, and aggressive behaviors (Anastasio et al., 2015). Morici et al. (2015) found that 5-HT2AR had been implicated in different neuropsychiatric and neurological disorders including schizophrenia, attention deficit hyperactive disorder, and Alzheimer’s disease. 5-HT2AR highly expressed in the apical dendrites of pyramidal neurons in layer 5 of the cortex suggests that cortical 5-HT2AR modulates cortical function *via* distinctive mechanisms and thus plays a key role in the modulation of different cortical functions (Jakab and Goldman-Rakic, 1998). Interestingly, 5-HT1AR and 5-HT2AR appear to be co-expressed in a large fraction of pyramidal cells in the medial prefrontal cortex (mPFC). Therefore, they may regulate, in a cooperative manner the way, pyramidal neurons to encode excitatory inputs into action potential firing. However, how this interaction affects behavior is still unknown (Morici et al., 2015).

However, only a few studies have addressed the direct effects of glucocorticoids on the 5-HT system. As we’ve known, 5-HT1AR and 5-HT2AR are two essential 5-HT receptors, the interaction in the 5-HT1AR and 5-HT2AR therefore likely has a significant role in modulating mentality, and has been confirmed to be closely related to ADHD associated behaviors. Takao’s research found that corticosterone treatment could decrease 5-HT1AR in the hippocampus but increase 5-HT2AR in the frontal cortex (Takao et al., 1997). Also, the study from Fairchild et al. (2003) found that chronic corticosterone treatment could induce the reduction in 5-HT1AR sensitivity in the dorsal raphe nucleus. These results suggested that the release of glucocorticoids resulting from the activation of the HPA axis may change the function of 5-HT neurons during stress. The GR system can modulate the symptoms of ADHD. GR is also involved in stress response in the 5-HT system (Higuchi et al., 2017). Thus, we reasoned that GR, 5-HT1AR and 5-HT2AR might be related. Since the exact relationship in the prefrontal cortex in ADHD was still unknown, we performed their correlation analyses to further validate their relationship in the ADHD model.

Based on the relationships between ADHD and GC/GR or the 5-HT system as discussed above, in this study, we proposed a hypothesis that GC/GR may influence ADHD through the 5-HT system. A series of behavioral assessments and biochemical analyses were used to explore the correlation between GC/GR and 5-HT. Spontaneously hypertensive rats (SHR) were chosen as the ADHD model and Wista Kyoto rats (WKY) were chosen as the normal control. We first explored the influence of glucocorticoid receptors on the 5-HT system in the ADHD model by behavioral assessments. Then, we obtained extracellular fluid from the prefrontal region of the living brains by microdialysis technique after localizing the prefrontal area by the brain stereotaxic apparatus, and measured the level of 5-HT by high-performance liquid chromatography (HPLC). The expressions of GR, 5-HT1AR and 5-HT2AR in the prefrontal cortex were analyzed through immunohistochemistry. Our designed experiments of GR agonist and inhibitor interventions were also employed to investigate the effect of prefrontal GC/GR function on the 5-HT system in the ADHD model.

## 2. Materials and methods

### 2.1. Animals

Both 3 weeks old male spontaneously hypertensive rats (SHR; *n* = 45) and Wista Kyoto rats (WKY; *n* = 15), weighing 50–60 g, purchased from Shrek Experimental Center (SPF, Shanghai, China), were acclimated in our animal facility for a period of a week and housed in plastic cages with a 12:12 h light-dark cycle (light from 7:00 to 19:00) under a constant temperature of 23–24°C and relative humidity of 50–60%. Throughout the experiments, the animals had free access to water and food. All the procedures were done in strict accordance with the guidelines of Animal Management Rules of the Ministry of Health of the People’s Republic of China (documentation Number 55, 2001, Ministry of Health of PR China) and the international guidelines on the ethical use of laboratory animals. The experiments were carried out with the approval of the Animal Ethics Committee of Fujian Medical University Union Hospital (FJMUIACUC2019-0105).

### 2.2. Drugs

**FIGURE 1:**
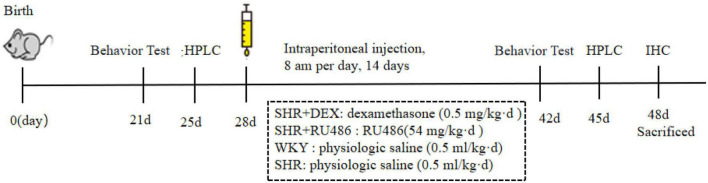
Schematic of experimental procedures.

To investigate the correlation between GC/GR and SHR, we used dexamethasone sodium phosphate (DEX) (HY-14648, MedChemExpress (MCE), Monmouth Junction, NJ, USA) as the GR agonist, and RU486 (HY-13683, MedChemExpress (MCE), Monmouth Junction, NJ, USA) as the GR antagonist for the intervention experiments. Starting at postnatal day 28 (PD28), after performing the behavioral tests, we randomly divided 45 SHR into three groups, 15 in each group, and treated them with GR agonist DEX (SHR + DEX group), GR inhibitor RU486 (SHR + RU486 group), and saline (control group, without drug intervention), respectively. According to the literature, rats in the SHR + DEX group were given a single intraperitoneal dose of dexamethasone (0.5 mg/kg per day) (Chunnv, 2013) at 8 am per day for 14 d. SHR + RU486 group was given RU486 54 mg/kg per day. WKY and SHR groups were administered with an equivalent of the body weight of physiologic saline (0.5 ml/kg per day). After behavioral assessments, the rats were sacrificed, and brain tissues were immediately collected for experiments or stored at −80°C for further analysis. The schematic of experimental procedures for behavioral tests, pharmacological intervention, and sacrificed time is in Figure 1.

### 2.3. Behavioral experiments

Open field test (OFT) and Làt maze test were performed for behavioral assessments. Both of these tests have been widely used in the assessment of ADHD-related behaviors in rodents, and the detailed methods were described as follows (Archer, 1973; Wu et al., 2010; Yin et al., 2014). OFT and Làt maze tests were conducted for all rats at the beginning and end of the 2-weeks drug administration period.

#### 2.3.1. OFT

The open field is a black box (60 × 60 × 40 cm), and in order to facilitate data processing, the total area was equally divided into 9 equal squares virtually (20 × 20 cm). Between trials, the floor of the box was wiped with 75% ethanol to remove scent marks. OFT was performed in a quiet room from 8:00 to 12:00. Each rat was placed in the center square of the field. The number of squares crossed (horizontal activity) and the rearing frequency (vertical activity, defined as the number of times the animals stood on their hind limbs) were recorded for analysis of locomotor activity. The rat behaviors were monitored for 5 min each time.

#### 2.3.2. Làt maze test

The Làt maze test was performed to assess non-selective attention (NSA). The Làt maze consisted of a 60 × 60 × 40 cm wooden box with a 30 × 30 × 40 cm transparent plastic box inserted in the middle, thus providing a 60 cm long, 15 cm wide, and 40 cm high corridor, where the rats could traverse freely (Sadile et al., 1988). The box was illuminated by a white, cold 4 W lamp placed 60 cm above the floor in the center of the cove. The box was located in a dark sound-attenuated room. The movements were tracked for 30 min and recorded by video cameras. The frequency of rearing episodes on the hind limbs of the rats was used to index NSA.

### 2.4. Determination of 5-HT in the prefrontal cortex

The concentrations of 5-HT in extracellular fluids in the prefrontal cortex of the rat brains, with or without drug intervention, were determined by HPLC. The rats were anesthetized before being fixed to the stereotactic apparatus (Stoelting, USA). The stereotaxic placement of the probes was according to the stereotaxic coordinates of the rat brain atlas (Paxinos and Watson, 2007). The region of the prefrontal cortex (in front of the anterior fontanelle *Y* = 3.7 mm, next to the parasagittal sinus *X* = 0.5 mm, under horizontal plane *Z* = 4 mm) was positioned, then the microdialysis guide tube was inserted and fixed, and the skin was sutured. After that, the respiration, heartbeat, reaction, and other life signs of rats were monitored for 2 h to ensure the rats were in a stable condition, and then the rats were placed in a cage and fed separately for 3 days. Three days later, the needle core of the guide tube was removed, and the CMA/12 microdialysis probe (CMA, Sweden) was implanted. CMA/12 microdialysis needle (4 mm long, 0.05 mm diameter) was connected to the CMA/102 microinjection pump and the CMA170 cold collector. The microinjection pump was constituted with artificial CSF and then perfused with artificial CSF at 37°C with a flow rate of 2 μL/min. Extracellular fluids (ECF) were collected every 15 min for 120 min and stored at –70°C until HPLC analysis.

For HPLC, an internal standard of 10 mg isopropyl norepinephrine (BCBN8508, Sigma, USA) was mixed with acetonitrile containing 2% formic acid (Sigma, USA), which was dissolved and diluted to a mass concentration of 10 ng/ml, and stored in 4°C. The 5-HT standard solution that was formulated in different concentrations, including upper limit and lower limit, was confirmed by gas chromatography technique 10 mg of samples dissolved in 10 mL of ultra-pure water were injected using a Water 464 autosampler (Water, USA) onto a GRACE HPLC reversed-phase column (250 mm × 3.9 mm 5.0 um) (Grace, USA) using acetonitrile: 2% formic acid (20: 80, v: v): 0∼2.5 min, acetonitrile: 2% formic acid (60: 40, v: v): 2.5∼4.0 min as the mobile phase at a flow rate of 0.4 ml/min, the balance time was 2 min; and determined by a photodiode array detector at the wavelength of 280 nm. The column temperature was kept at 25°C. HPLC data acquisition and analysis were managed by Waters-Empower software. Each sample was injected in the amount of 20 μL by using an autosampler five times. The relative standard deviations (RSDs) were then calculated (Inoue et al., 2000).

The relative recovery is defined as the ratio of the concentration of analyses to the concentration of the same substance in the medium (not in the dialysis probe) and is used to assess the efficiency of probe dialysis. We calculated the relative recovery (R) of probes *in vitro* by calculating the ratio of the chromatographic peak area (Am) of 5-HT in a microdialysis sample *in vitro* to the peak area (As) of a standard solution: *R* = Am/As × 100.

### 2.5. Immunohistochemistry

The expressions of 5-HT and GR in the prefrontal cortex of the rats were measured by immunohistochemistry. The prefrontal cortex was briefly harvested from each animal and fixed in 4% paraformaldehyde (Anatech, Battle Creek, Michigan) at 4°C for 24 h. The perfused brains were processed for paraffin embedding. Brains were sectioned on the microtome at 3 μm thickness and mounted on polyionic slides.

Tissue sections were stained with anti-5-HT1AR antibody diluted 1:100 (BA1391, Dianguo NeoBioscience company, Beijing, China), and anti-5-HT1AR antibody diluted 1:100 (BA2048, Dianguo NeoBioscience company, Beijing, China), kept overnight at 4°C temperature, and then detected with S-A/HRP Polymer conjugated biotinylated secondary antibody (1:600; SP9001, Zhongshan Jinqiao NeoBioscience company, Beijing, China). Tissue sections were stained with anti-GR antibody diluted 1: 500 (ab183127, Abcam company, USA), and detected with PV-9002 (Zhongshan Jinqiao NeoBioscience company, Beijing, China). DAB chromogen (Zhongshan Jinqiao NeoBioscience Company, Beijing, China) was used as a peroxidase substrate. Slides were counterstained with hematoxylin, and dehydrated.

Negative control tests were carried out using 0.1M Phosphate Buffered Saline (PBS) (Zhongshan Jinqiao NeoBioscience Company, Beijing, China) instead of primary antibody in order to establish the specificity of the immunostaining. The negative control tissues contained no 5-HT1AR, 5-HT2AR, or GR immunoreactivity in any structure.

The expressions of 5-HT1AR, 5-HT2AR, and GR were measured by immunohistochemistry and assessed using quantitative integrated optical density (IOD). Five sections were selected from the prefrontal region of each rat in each group, and five non-overlapping fields of view were randomly selected in each section and photographed at 40 × 10 magnification. The presence of brown or tan particles in the cells was taken as a positive signal, and then we calculated the sum of the mean optical density per positive cell in the prefrontal area and compared the mean IOD SUM per section. The IOD SUM of immunohistochemical staining was determined for each immunohistochemical section at 40 × 10 magnification using an image analysis system (Image-Pro Plus 6.0 software, Media Cybernetics, Silver Spring, MD, USA). All samples were evaluated independently by two investigators. In the event of inconsistent statistical results, the statistical evaluation would be repeated.

### 2.6. Statistical analysis

All statistical analyses and figures were obtained by IBM SPSS statistics 21.0 for Windows (SPSS, Chicago, IL, USA) and GraphPad Prism 8.0 software (GraphPad Software, CA, USA). The data were shown as mean ± SEM. The difference between the groups was evaluated using one-way analysis of variance (ANOVA) followed by the *post hoc* with Tukey test. Pearson correlation analysis was used for correlation analysis. *p* < 0.05 was considered statistically significant, and all *p* values were two-sided.

## 3. Results

### 3.1. Behavioral tests

#### 3.1.1. Open field test (OFT)

**FIGURE 2:**
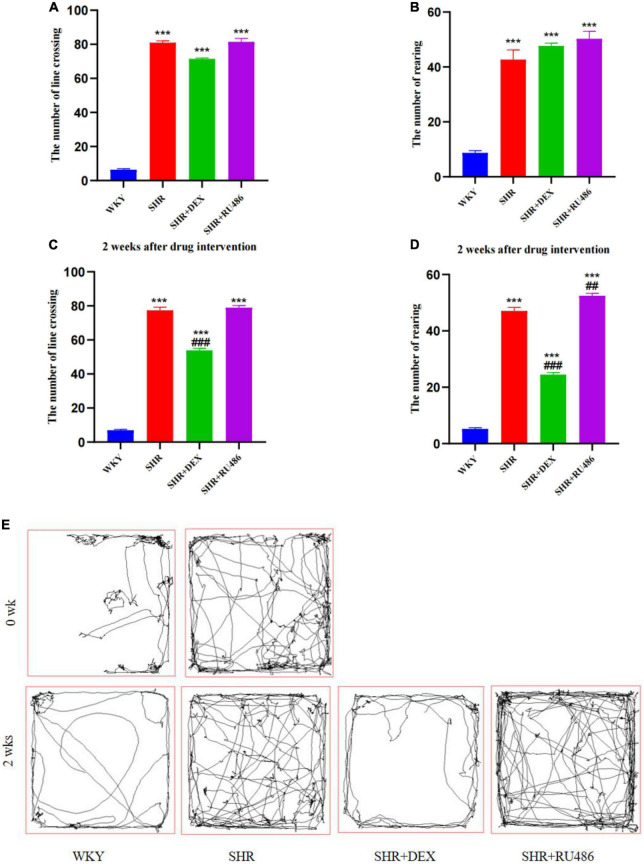
Behavioral performance in open field test (OFT) (*n* = 15 in each group). **(A)** The number of line crossings before drug intervention, **(B)** the number of rearing before drug intervention, **(C)** the number of line crossing after drug intervention for 2 weeks, **(D)** the number of rearing after drug intervention for 2 weeks, **(E)** representative pathways of each group before and after drug intervention. One-way analysis of variance (ANOVA) with repeated measures followed by *post hoc* with Tukey test. All data are presented as mean ± SEM. ***p < 0.001, compared to Wista Kyoto rats (WKY) group; ##*p* < 0.01, ###*p* < 0.001, compared to spontaneously hypertensive rats (SHR) group.

To evaluate the behavior of spontaneous locomotor activity and impulsivity in SHR and WKY rats, OFT was performed. As shown in Figures 2A, B, the results revealed that both the number of square crossings and rearing of SHR were significantly higher than those of WKY rats (*p* < 0.001, Figures 2A, B).

After 2 weeks of drug intervention, SHR + DEX group displayed a decrease in both line crossing (53.87 ± 1.171) and rearing (24.47 ± 0.780), compared to SHR group (77.40 ± 1.167 and 47.07 ± 1.278, respectively) (*p* < 0.001, Figures 2C, D). In addition, SHR + RU486 group (52.47 ± 0.883) showed more rearings than SHR group (47.07 ± 1.278) (*p* < 0.001, Figure 2D). There was no significant difference in the numbers of line crossings between SHR + RU486 and SHR groups (*p* > 0.05), but both of the groups showed more line crossings than WKY group (*p* < 0.001, Figure 2C). The representative movement pathways of each group before and after drug intervention are shown in Figure 2E.

#### 3.1.2. Làt maze

**FIGURE 3:**
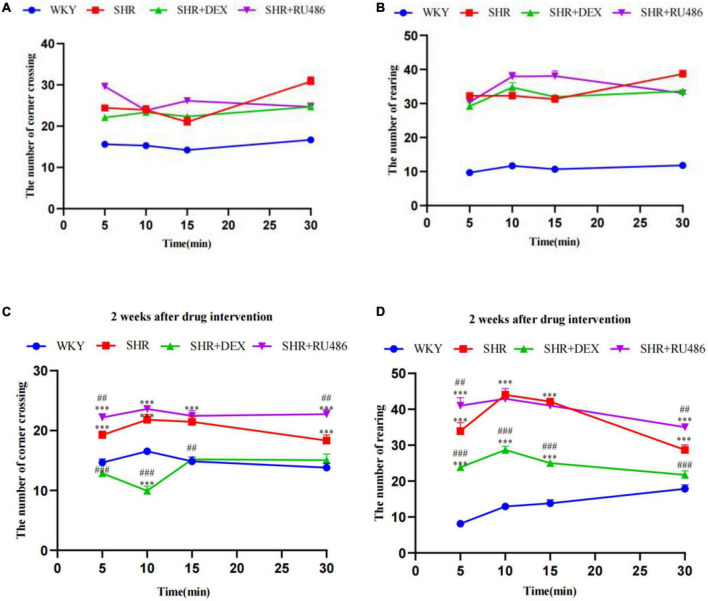
Behavioral performance in Làt maze (*n* = 15 in each group). **(A)** The number of corner crossings before drug intervention, **(B)** the number of rearings before drug intervention, **(C)** the number of corner crossings after drug intervention for 2 weeks, **(D)** the number of rearings after drug intervention for 2 weeks. One-way analysis of variance (ANOVA) with repeated measures followed by *post hoc* with Tukey test. All data are presented as mean ± SEM. ^***^p < 0.001, compared to Wista Kyoto rats (WKY) group; ##*p* < 0.01, ###*p* < 0.001, compared to spontaneously hypertensive rats (SHR) group.

In order to assess the degree of attention deficit in SHR, Làt maze was performed. Hyperactivity levels were determined by the number of crossing corners, and the number of rearing was used to assess the level of non-selective attention in visuospatial attention. During the 30 min Làt maze test, both the number of crossing corners and the number of rearing in SHR group were significantly high than in WKY group (*p* < 0.001, Figures 3A, B), suggesting hyperactive and attention-deficit behaviors in SHR.

However, after 2 weeks of drug intervention, both the numbers of crossing corners and rearing in SHR + DEX group were significantly less than SHR + RU486 group and SHR group (*p* < 0.001, Figures 3C, D). Meanwhile, SHR + RU486 group had more crossing corners when compared to SHR group (*p* < 0.001, Figure 3C). Similarly, SHR rats crossed more corners and reared more than WKY rats (*p* < 0.001). These results suggested that the GR agonist might lessen the hyperactive and attention-deficit behaviors in SHR.

### 3.2. The 5-HT levels in the prefrontal cortex of rats

**FIGURE 4:**
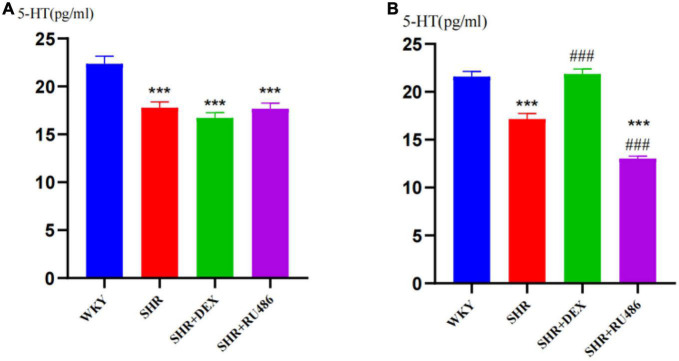
Levels of 5-hydroxytryptamine (5-HT) in the prefrontal cortex of rats in four groups (*n* = 15 in each group) before and after drug intervention. **(A)** Pre-intervention and **(B)** post-intervention content of 5-HT in the extracellular liquids of the rat prefrontal cortex, respectively. One-way analysis of variance (ANOVA) with repeated measures followed by *post hoc* with Tukey test. All data are presented as mean ± SEM. ***p < 0.001, compared to Wista Kyoto rats (WKY) group; ###*p* < 0.001, compared to spontaneously hypertensive rats (SHR) group.

To further evaluate the levels of 5-HT in the prefrontal cortex of rats before and after GR agonist or inhibitor interventions, we collected the extracellular fluids from the prefrontal areas by a brain stereotaxic apparatus and microdialysis technique, and measured the 5-HT concentrations by HPLC. As shown in Figure 4A, the expression levels of 5-HT in all SHR groups were significantly lower than those in the WKY group (*F* = 15.78, *p* < 0.001, Figure 4A). After 2 weeks of drug intervention, the expression level of 5-HT in the prefrontal cortex of SHR + DEX group (21.87 ± 0.506) was significantly higher than that in the SHR group (17.16 ± 0.589) (*p* < 0.001), while the RU486 group (13.03 ± 0.250) was significantly lower than the SHR group (*p* < 0.001, Figure 4B). These results indicated that ADHD rats have reduced 5-HT levels in the prefrontal cortex and suggested that the GR agonist elevated the expression of 5-HT in the prefrontal cortex of SHR, whereas the GR inhibitor reduced the expression of 5-HT.

### 3.3. The expression of GR in the prefrontal cortex of rats

**FIGURE 5:**
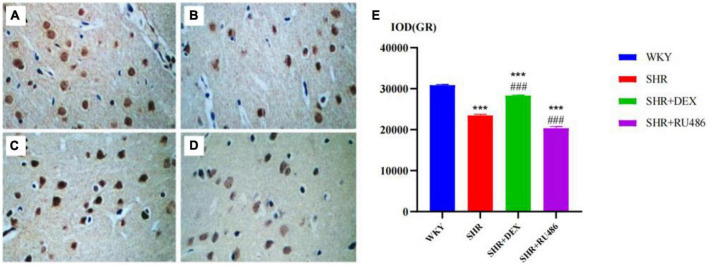
The immunohistochemical images for the measurements of glucocorticoid receptor (GR) levels in the prefrontal cortex of rats in four groups (× 400) [**(A)** Wista Kyoto rats (WKY) group; **(B)** spontaneously hypertensive rats (SHR) group; **(C)** SHR + dexamethasone (DEX) group; **(D)** SHR + RU486 group); Comparison of GR levels in the prefrontal cortex of rats in four groups **(E)**. One-way analysis of variance (ANOVA) with repeated measures followed by *post hoc* with Tukey test. All data are presented as mean ± SEM. ***p < 0.001, compared to WKY group; ###*p* < 0.001, compared to SHR group.

In order to illuminate the differences in GR expression in the prefrontal cortex of SHR and WKY rats, we used the immunohistochemical method to measure the expression levels of GR. The results confirmed the differences in the expression of GR in the prefrontal cortex of SHR and WKY rats, as shown in Figure 5. The expression level of GR in the SHR groups (Figure 5B) was lower than in the WKY group (Figure 5A), and the difference was statistically significant (*F* = 319.2, *p* < 0.001). Compared with the SHR group (23480 ± 259.3), (Figure 5B) the expression level of GR in the SHR + DEX group (28308 ± 150.7) (Figure 5C) was significantly higher (*p* < 0.001), while the RU486 group (Figure 5D) (20347 ± 403.2) was significantly lower (*p* < 0.001, Figure 5E). These findings suggested that the GR deficiency is most likely associated with ADHD pathogenesis.

### 3.4. The expression of 5-HT receptor in the prefrontal cortex of rats

**FIGURE 6:**
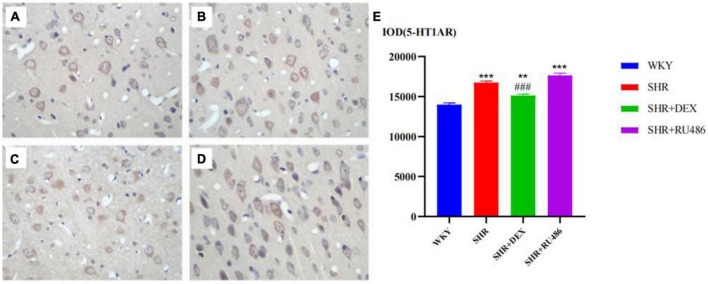
Comparison of 5-HT1A receptor (5-HT1AR) levels in the prefrontal cortex of rats in four groups (× 400) [**(A)** Wista Kyoto rats (WKY) group; **(B)** spontaneously hypertensive rats (SHR) group; **(C)** SHR + dexamethasone (DEX) group; **(D)** SHR + RU486 group]; Comparison of 5-HT1AR levels in the prefrontal cortex of rats in four groups **(E)**. One-way analysis of variance (ANOVA) with repeated measures followed by *post hoc* with Tukey test. All data are presented as mean ± SEM. ***p < 0.001, **p < 0.01, compared to WKY group; ###*p* < 0.001, compared to SHR group.

We also used the immunohistochemical assay to further compare the expression levels of 5-HT receptors in SHR and WKY rats. The results showed that the expression level of 5-HT1AR in the SHR group was higher than in the WKY group (*p* < 0.001). The expression level of 5-HT1AR in the SHR + DEX group was lower than that in the SHR group (*p* < 0.001) but higher than in the WKY group (*p* < 0.01). Similarly, the level of 5-HT1AR in the SHR + RU486 group was higher than WKY (*p* < 0.001), but no difference was found between the SHR + RU486 group and SHR group (Figure 6). The results demonstrated that the expression of 5-HT1AR in the prefrontal cortex of SHR was upregulated, but the GR agonist intervention reduced the expression of 5-HT1AR.

**FIGURE 7:**
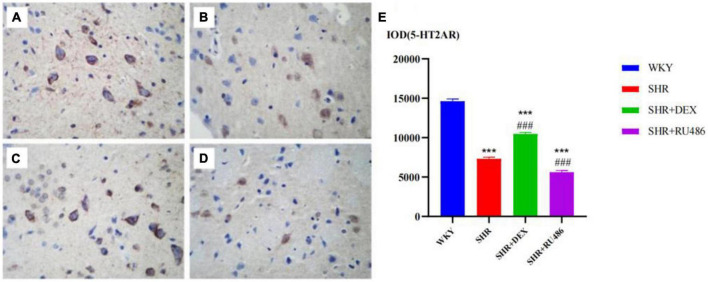
Comparison of 5-HT1A receptor (5-HT2AR) levels in the prefrontal cortex of rats in four groups (× 400) [**(A)** Wista Kyoto rats (WKY) group; **(B)** spontaneously hypertensive rats (SHR) group; **(C)** SHR + dexamethasone (DEX) group; **(D)** SHR + RU486 group]; Comparison of 5-HT2AR levels in the prefrontal cortex of rats in four groups **(E)**. One-way analysis of variance (ANOVA) with repeated measures followed by *post hoc* with Tukey test. All data are presented as mean ± SEM. ***p < 0.001, compared to WKY group; ###*p* < 0.001, compared to SHR group.

We also found that the expression level of 5-HT2AR in the WHY group was the highest among the groups (*p* < 0.001). The expression level of 5-HT2AR in the SHR + DEX group was significantly higher than in the SHR group (*p* < 0.001), while the expression level of 5-HT2AR in the SHR + RU486 group was lower than in the SHR group (*p* < 0.001, Figure 7). This result suggested that the expression of 5-HT2AR in the prefrontal cortex of SHR was downregulated, but it could be increased by the intervention of a GR agonist or further decreased by the intervention of a GR inhibitor.

### 3.5. Correlation analysis of GR and 5-HTAR expression in the prefrontal cortex

**FIGURE 8:**
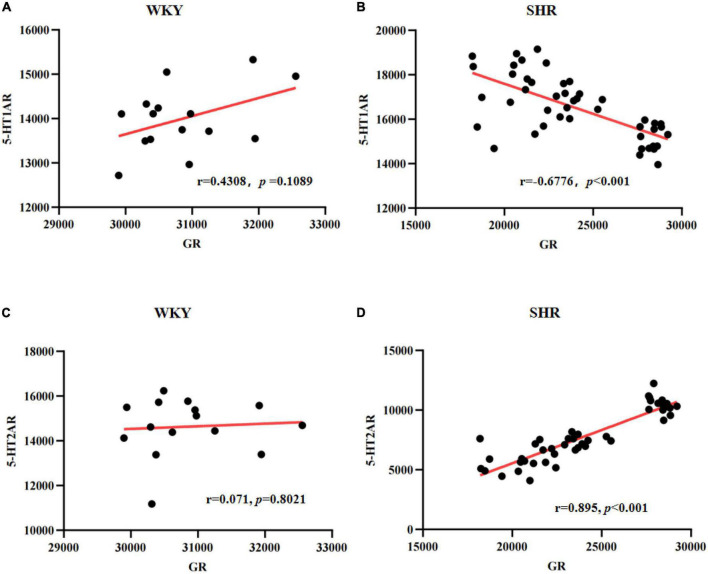
Scatter plots for the correlation between glucocorticoid receptor (GR) and 5-HT1A receptor (5-HT1AR) or 5-HT2AR in spontaneously hypertensive rats (SHR) or Wista Kyoto (WKY) rats: Correlation between GR and 5-HT1AR in WKY **(A)** or SHR **(B)**; Correlation between GR and 5-HT2AR in WKY **(C)** or SHR **(D)**.

As known, 5-HT1AR and 5-HT2AR are two essential 5-HT receptors, and the interaction between the 5-HT1AR and 5-HT2AR therefore likely has a significant role in modulating mentality and has been confirmed to be closely related to ADHD-associated behaviors. To further explore the correlation between GR and 5-HT receptor expressions, we employed Pearson correlation analysis. As shown in Figures 8A, C, in the prefrontal cortex of WKY rats, there was no correlation between the expressions of GR and 5-HT1AR (*r* = 0.4308, *p* = 0.1089), nor between the expressions of GR and 5-HT2AR (*r* = 0.071, *p* = 0.8021). However, in SHR, the results showed that the quantitative integrated optical density (IOD) of GR and 5-HT1AR in the prefrontal cortex was consistent with the bivariate normal distribution (Figure 8B). Correlation analysis between GR and 5-HT1AR showed r was −0.6776 (*p* < 0.001), suggesting that they were negatively correlated (Figure 8B). The IOD of GR and 5-HT2AR in the prefrontal cortex was consistent with the bivariate normal distribution (Figure 8D). Correlation analysis between GR and 5-HT2AR showed r was 0.895 (*p* < 0.001), suggesting that they were positively correlated (Figure 8D). The results further suggested that GR may inhibit the expression of 5-HT1AR and promote the expression of 5-HT2AR, and all 5-HT related receptors may be associated with ADHD pathogenesis.

## 4. Discussion

Attention-deficit hyperactivity disorder (ADHD) is a common disorder that affects learning, social relationships, and the quality of life of the individual and leads to familial and societal burdens. Despite the increased prevalence of the disorder, the pathogenesis of ADHD is poorly understood. Thus, there is an immediate need to study the etiology and precise pathogenesis mechanism of ADHD. In this study, through systematic and quantitative analysis, we explored the roles that GR and the 5-HT system play in ADHD and the correlation between GR and the 5-HT system.

We first reported the alterations of behaviors before and after drug intervention according to the behavioral test. Results confirmed the effectiveness of SHR as a model animal for ADHD. After GR agonist intervention, the attention deficit and hyperactive behaviors of SHR were ameliorated by calculating the numbers of square crossing and rearing in OFT. Similarly, in the Làt maze, mitigation of the non-selective attention deficit behavior of SHR was observed by calculating the numbers of corners crossing and rearing. After GR inhibitor intervention, the attention deficit and hyperactive behaviors of SHR were elevated. These findings showed that GR agonists helped correct the hyperactive and attention-deficit behaviors in SHR, and GR may be closely related to the onset of ADHD. This suggested that the diminished GR expression and/or function might have been a potential cause of deficient cortisol feedback, making the activity of the HPA axis decrease under stress, and the SHR manifested a disturbed HPA axis function and hypocortisol response. Lu et al. (2021) found that the absence of GR in astrocytes induced depressive-like behaviors, but restoring astrocytic GR expression in the medial PFC prevented the phenotype. Wu et al. (2017) and our research found that children with ADHD had a reduction in serum cortisol, and the SHR also had a reduction in serum corticosterone (Chen et al., 2012). Our research found that dysfunction in the HPA axis was generally considered to be responsible for low blood cortisol concentrations, which in turn might be related to the core ADHD symptoms of attention deficit, hyperactivity, and impulsive behaviors (Ma et al., 2011). Barsegyan et al. (2019) found that GR enhanced recognition memory *via* basolateral amygdala-driven facilitation of prelimbic cortex interactions. Li et al. (2021) also found that with the treatment of GR agonist Dex, the rats under chronic unpredictable mild stress ameliorated the depression-associated behavior. Wang et al. (2020) found that the rats presented depression-and anxiety-like behavior after intra-hippocampal infusion of the GR inhibitor RU486. In SHR, elevated expression of Nr3C1 in the PFC and hippocampus enhanced the sensitivity of the response to glucocorticoid negative feedback and reduced the release of cortisol (Sullivan and Gratton, 2002; Anacker et al., 2011). Children with ADHD have a blunted cortisol response to psychosocial stressors, a decreased cortisol awakening response, and a lower plasma daytime cortisol concentration. Family-based association tests indicated that the Nr3C1 single-nucleotide polymorphism was associated with HPA axis reactivity (Wust et al., 2004) and the ADHD morbidity rate (Fortier et al., 2013). All the findings suggest that the GR system can modulate the symptoms of ADHD, and they are in accordance with our findings.

Many have explored the relationship between the 5-HT system and behavioral disorders. For example, Dalley and Roiser (2012) found that the CNS inhibitory neurotransmitter 5-HT correlated with impulse control and emotions. van der Meer et al. (2016) found that the serotonin transporter gene polymorphism 5-HTTLPR is known to influence the relationship between stress exposure and ADHD severity. A study indicated that the peripheral function of the 5-HT metabolic pathway can serve as a significant predictor of aggressive behavior (Comai et al., 2016). Roessner et al. (2009) found reduced brain serotonin function in SHR and a higher density of serotonin transporters in the striatum, compared to control WKY rats. In 5-HT depletion mice, male mice displayed increased impulsivity and high aggression levels (Mosienko et al., 2012; Gutknecht et al., 2015) and female mice displayed increased emotional reactivity to aversive conditions (Gutknecht et al., 2015). Miczek et al. (2015) found that aggressive mice with short attack latencies have an elevated 5-HT1A receptor activity as well as reduced serotonin levels. Importantly, many studies have found that children with ADHD symptoms had lower 5-HT levels compared with healthy controls (Spivak et al., 1999; Banerjee and Nandagopal, 2015; Wang et al., 2018). As expected, in this study, the quantitative results showed that the levels of 5-HT in the prefrontal cortex in SHR were decreased, consistent with the literature. Taken together, the characteristics of ADHD, such as impulsivity, temper tantrums, or aggression, may correlate to the lower level of peripheral 5-HT, and treatments by increasing 5-HT levels have been effective in mitigating these symptoms.

To explore the correlation of GC/GR with 5-HT, we investigated the behaviors of SHR and the levels of 5-HT in the prefrontal cortex. Results showed that the GR agonist DEX not only increased the expression of GR, but also increased the level of 5-HT in the prefrontal cortex and reduced the attention deficit and hyperactivity behavior in SHR. The corresponding changes between GR and 5-HT suggested that GR might play a vital role in the pathogenesis of ADHD through 5-HT.

The abnormal expression of 5-HT receptors might also be related to the pathogenesis of ADHD. It is known that the prefrontal cortex of ADHD children is impaired. The prefrontal cortex is rich in 5-HT receptors 5-HT1AR and 5-HT2AR (Chalmers and Watson, 1991; Gao et al., 2018). These two receptors exert, in the mPFC, opposite effects on neuronal activity. Meijer and de Kloet (1994) found that GC can regulate gene expression by binding to the GC response element in the promoter region of the 5-HT1AR gene, inhibiting the 5-HT1AR gene expression in rats. Drevets et al. (2007) found that the reduction in 5-HT1AR mRNA expression in depression might arise secondarily to cortisol hypersecretion, as the 5-HT1AR mRNA expression and density are under tonic inhibition by GR stimulation. This suggested that there might be a correlation between GC/GR and 5-HT1AR. Lanfumey et al. (2008) also found that chronic GC administration or chronic contact stress downregulated 5-HT1AR in the cortex or hippocampus, but with a long-term low level of GC, 5-HT1AR was upregulated, which resulted in a decreased decision-making ability of emotion regulation behavior, and a weak self-examination and self-monitoring consciousness. In our study, we found an increase in 5-HT1A receptor expression and a decreased expression of prefrontal GR in SHR (Figure 7B), consistent with the previous work. After DEX intervention, the level of 5-HT increased with a decrease in 5-HT1AR and ameliorated attention deficit and hyperactive behaviors, suggesting that the GR agonist or 5-HT1AR inhibitor may serve as a potential target for the treatment of ADHD.

Moreover, the demonstration of 5-HT2AR is of great relevance for schizophrenia, and 5-HT2AR antagonists represent major drugs in the treatment of schizophrenia. Borroto-Escuela et al. (2021) research showed that there was a reduction of postjunctional 5-HT1AR protomer signaling in the forebrain upon activation of the 5-HT2AR protomer in depression. 5-HT2AR is one of the principal post-synaptic receptors for 5-HT, which is localized in the cortex, ventral striatum, hippocampus, and amygdala (López-Giménez et al., 2001). 5-HT2AR gene polymorphism is associated with impulsiveness and ADHD. Trajkovska et al. (2009) found that the 5-HT2AR protein expression increased in the hippocampus of transgenic mice with overexpression of GR, and the binding capacity of 5-HT2AR in the dorsal hippocampus was especially increased; however, the expression level of 5-HT2AR protein, especially in the frontal lobe, in the hippocampus of mice with low GR expression was decreased. The culture of hippocampal tissue *in vitro* showed that GC intervention could promote the expression of 5-HT2AR, which could be blocked by RU486 and spironolactone (Trajkovska et al., 2009). It was suggested that the activation of GR upregulated the level of 5-HT2AR, while the decrease in GR function downregulated the level of 5-HT2AR. Our study found that the expression of 5-HT2AR in the prefrontal region of SHR was decreased, consistent with the literature; GR agonists could increase the expression of 5-HT2AR in the prefrontal area of SHR.

Researchers believe that the functionally opposing effects of the postsynaptic 5-HT1A and 5-HT2A receptors have resulted in a disturbed balance in the 5-HT system that may contribute to the pathophysiology of depression, anxiety, and impulsivity. Obviously, our research found that the interaction of the 5-HT and GR systems together influenced ADHD behaviors in SHR. In SHR, the low level of GR resulted in an upregulation of 5-HT1AR expression and a dysregulation of 5-HT2AR expression, which also decreased the level of 5-HT in the prefrontal area. It further disrupted the balance of monoamine neurotransmitter metabolic processes and thereby caused hyperactivity and attention deficit behaviors in SHR. According to our results, we infer that both the 5-HT1AR and 5-HT2AR might be involved in ADHD, and they can serve as novel targets for drug development to improve ADHD treatments. These findings suggest a potential molecular pathway mediating the effects of glucocorticoid and the 5-HT system in ADHD behaviors. Nevertheless, this work only confirmed the correlations among 5-HT1AR, 5-HT2AR, and GR in ADHD at this stage, and it is certainly important to reveal the mechanism of ADHD pathogenesis at a molecular level and to help the discovery of new drug targets for the prevention and treatment of ADHD in the future.

## 5. Conclusion

In summary, our results clarified that SHR were dysregulated in the central 5-HT system, which was characterized by a lack of 5-HT content in the prefrontal region, increased expression of 5-HT1AR, and decreased expression of 5-HT2AR. By activating GR, the level of 5-HT in the prefrontal area increased, along with downregulation of 5-HT1AR expression and upregulation of 5-HT2AR expression, thus mitigating the hyperactivity and attention deficit behaviors in SHR. Apparently, it is a necessity to recover this balance for therapeutic needs. Drugs for upregulation of 5-HT2AR or downregulation of 5-HT1AR may be effective in ADHD.

## Data availability statement

The original contributions presented in this study are included in the article/supplementary material, further inquiries can be directed to the corresponding author.

## Ethics statement

All animal experiments in this research complied with the ARRIVE guidelines and were carried out following the National Institutes of Health Guide for the Care and Use of Laboratory Animals (NIH eighth edition, revised 2011) and Institutional Animal Care. The Animal Ethics Committee approved all procedures of Fujian Medical University Union Hospital.

## Author contributions

XL performed the statistical analysis and wrote the manuscript. All authors conceived of the study and participated in its design as well as coordination, helped to revise the manuscript, read, and approved the final manuscript.
